# Establishment of sex-specific predictive models for critical illness in Chinese people with the Omicron variant

**DOI:** 10.3389/fmicb.2023.1224132

**Published:** 2024-01-23

**Authors:** Rui Yang, Xin Guan, Ziguang Niu, Rulin Zhang, Siang Lv, Xiang Xu, Yingying Zhao, Jun Wu

**Affiliations:** ^1^Department of Laboratory Medicine, Shanghai General Hospital, Shanghai Jiao Tong University School of Medicine, Shanghai, China; ^2^Department of Laboratory Medicine, Jiading Branch of Shanghai General Hospital, Shanghai Jiao Tong University School of Medicine, Shanghai, China; ^3^Department of Pathology, The Affiliated Hospital of Youjiang Medical University for Nationalities, Baise, China; ^4^Department of Medical Affairs, Shanghai General Hospital, Shanghai Jiao Tong University School of Medicine, Shanghai, China

**Keywords:** Omicron, predictive models, critical illness, COVID-19, sex-specific

## Abstract

**Introduction:**

The Omicron variant has rapidly spread throughout the world compared to the Delta variant and poses a great threat to global healthcare systems due to its immune evasion and rapid spread. Sex has been identified as a factor significantly associated with COVID-19 mortality, but it remains unclear which clinical indicators could be identified as risk factors in each sex group and which sex-specific risk factors might shape the worse clinical outcome, especially for Omicrons. This study aimed to confirm the relationship between sex and the progression of the Omicron variant and to explore its sex-biased risk factors.

**Methods:**

We conducted a retrospective study including 1,132 hospitalized patients with the COVID-19 Omicron variant from 5 December 2022 to 25 January 2023 at Shanghai General Hospital, and the medical history data and clinical index data of the inpatients for possible sex differences were compared and analyzed. Then, a sex-specific Lasso regression was performed to select the variables significantly associated with critical illness, including intensive care unit admission, invasive mechanical ventilation, or death. A logistic regression was used to construct a sex-specific predictive model distinctively for the critical illness outcome using selected covariates.

**Results:**

Among the collected 115 clinical indicators, up to 72 showed significant sex differences, including the difference in merit and the proportion of people with abnormalities. More importantly, males had greater critical illness (28.4% vs. 19.9%) and a significantly higher intensive care unit occupancy (20.96% vs. 14.49%) and mortality (13.2% vs. 4.9%), and males over 80 showed worse outcomes than females. Predictive models (AUC: 0.861 for males and 0.898 for females) showed 12 risk factors for males and 10 for females. Through a comprehensive sex-stratified analysis of a large cohort of hospitalized Omicron-infected patients, we identified the specific risk factors for critical illness by developing prediction models.

**Discussion:**

Sex disparities and the identified risk factors should be considered, especially in the personalized prevention and treatment of the COVID-19 Omicron variant.

## Introduction

Omicron was first identified and reported as a novel variant of severe acute respiratory syndrome coronavirus 2 (SARS-CoV-2) by the World Health Organization (WHO) on 24 November 2021^1^. The Omicron variant has rapidly spread throughout the world compared to the Delta variant and poses a great threat to global healthcare systems due to its immune evasion and rapid spread. Mohsin et al. found that people who were fully vaccinated and previously infected were several times more likely to be infected with Omicron than Delta or other variants (Mohsin and Mahmud, 2022). Omicron mutations spread rapidly over a short period of time, leading to unusual COVID-19 outbreaks in some parts of the world. Although Omicron is less severe than non-Omicron variants due to high infection rates and immune escape, it has overburdened the healthcare system. It has been reported that the lower severity of Omicron is a coincidence, as the ongoing rapid antigenic evolution is likely to produce new variants that could escape immunity and be more threatening, even when people have taken the full course of the COVID-19 vaccination (Markov et al., 2022).

A primary assessment of SARS-CoV-2 variants circulating in Shanghai from 15 November to 15 December 2022, indicated that the Omicron variant was predominant, including four notable Omicron lineages (BF.7, BA.5.2, XBB, and BQ.1) (Lu et al., 2023). From 1 December 2022 to 27 April 2023, 35,089 valid SARS-CoV-2 genome sequences from domestic cases in China were reported by the Chinese Center for Disease Control and Prevention; all 134 lineages were Omicron variants. The predominant lineages are BA.5.2 and its descendant (62.2%) and BF.7 and its descendant (32.3%). The predominant lineages in Shanghai were BF.7.14, BA.5.2.48, and DY.2 (Chinese Center for Disease Control and Prevention, 2023). This study mainly conducted a retrospective cohort study on the Omicron variant after the normalization of the national epidemic.

Sex-specific differences have been shown in clinical characteristics and prognosis for the COVID-19 Delta variant and other before variants in some retrospective studies (Alwani et al., 2021; Huang et al., 2021; Vahidy et al., 2021; Viveiros et al., 2022), in which sex differences in clinical characteristics, comorbidities, treatments, and outcomes were observed, as well as differences in risk predictors for critical illness. In addition to the high mortality rate, males also tended to have longer hospital stays, higher rates of hospitalization, ICU occupancy, secondary bacterial infections, shock, endotracheal intubation, and mechanical ventilation. Other earlier studies also found that males had higher CRP, erythrocyte sedimentation rate (ESR), alanine and aspartate aminotransferase, gamma-glutamyl transferase, ferritin, fibrinogen, lactate dehydrogenase (LDH), and activated partial thromboplastin time (APTT) than females (Meng et al., 2020; Ten-Caten et al., 2021). One of the causes of sex differences is the ACE-2 receptor gene on the X chromosome (Xp22.2), which is known as the receptor for the entry of SARS-CoV-2 into alveolar type 2 pneumocytes and is characteristically higher in females (Carrel and Willard, 2005; Hoffmann et al., 2020). This conditionally supports the reason why males are less protected. Another important factor related to the cause of the sex differences is the increased expression of TMPRSS2 in males (Bienvenu et al., 2020) through androgen receptors (ARs) (Wambier and Goren, 2020) because of the higher expression of ARs in males than in females (Rastrelli et al., 2021).

However, most of these conclusions are based on retrospective studies of the previous mutant variants. To date, few articles have been reported on the age and sex differences in the clinical characteristics, severity, or outcome of the Omicron variant, and it remains unclear which clinical indicators could be identified as risk factors in different sex groups and which sex-specific risk factors might shape the clinical outcome, especially for Omicrons. Therefore, this study aimed to confirm the relationship between early sex-specific clinical indicators and the critical illness outcome of the Omicron variant by selecting the risk factors to best explain the development of critical illness, including the conditions of intensive care unit admission, invasive mechanical ventilation, or death, through two distinctive predictive models for both sexes. We highly emphasize sex-specific differences in the clinical characteristics and prognosis of critical illness in patients with the Omicron variant, similar to the sex bias previously reported in other COVID-19 variant-related studies for preventive strategies and better public health services.

## Materials and methods

### Study cohort and design

We conducted a retrospective study including 1,132 hospitalized patients who tested positive for nuclear antigen or laboratory nucleic acid for COVID-19 from 5 December 2022 to 25 January 2023 at Shanghai General Hospital, including the North Branch, South Branch, and West Branch. In addition, we conducted next-generation sequencing (NGS) on 98 SARS-CoV-2 positive specimens and found that all the specimens examined were Omicron variants, among which BF.7 and BA.5.2 were the main lineages (detailed lineages are shown in Table S1). Given the urgent need to collect clinical data, the Ethics Committee of Shanghai General Hospital approved the study and agreed to waive informed consent. In this retrospective study, data collection was carried out independently by three fully trained personnel who collected the general characteristics (age, BMI, hospital days, etc.), clinical symptoms or signs, and laboratory indicators of the patient’s first admission from electronic medical records (shown in Tables 1, 2). All of the laboratory examinations in this study were performed according to the patients’ clinical care needs, and the radiologic assessments included either chest radiography or computed tomography (CT).

**Table 1:** Sex-specific general characteristics of 1,132 hospital patients.

	All (*n* = 1,132)	Male (*n* = 725)	Female (*n* = 407)	Value of *p*
Age [years, median (IQR)]	75.00 [66.00, 84.00]	71.7 [67.00, 84.00]	69.6 [63.25, 84.75]	0.134
0–49	127 (11.2)	70 (9.7)	57 (14.0)	0.032^*^
50–59	58 (5.1)	35 (4.8)	23 (5.7)	0.637
60–69	202 (17.9)	122 (16.8)	80 (19.7)	0.258
70–79	322 (28.5)	222 (30.6)	100 (24.6)	0.038^*^
≥80	422 (37.3)	276 (38.1)	146 (36.0)	0.523
BMI [body mass index, median (IQR)]	23.44 [20.76, 25.83]	23.80 [21.01, 25.83]	22.89 [20.35, 25.76]	0.178
Obesity (BMI ≥25)	311 (32.8%)	200 (32.8%)	111 (32.8%)	1
Comorbidities	937 (84.1%)	620 (86.7%)	317 (79.4%)	0.002^*^
Hypertension	604 (53.4%)	407 (56.1%)	197 (48.4%)	0.015^*^
Diabetes	342 (30.2%)	220 (30.3%)	122 (30.0%)	0.95
Malignancy	102 (9.0%)	68 (9.4%)	34 (8.4%)	0.638
Lung disease	148 (13.1%)	106 (14.6%)	42 (10.3%)	0.049^*^
Cardiovascular disease	480 (42.4%)	327 (45.1%)	153 (37.6%)	0.017^*^
Chronic liver disease	50 (4.4%)	30 (4.1%)	20 (4.9%)	0.646
Chronic kidney disease	95 (8.4%)	71 (9.8%)	24 (5.9%)	0.031^*^
Autoimmune disease	19 (1.7%)	7 (1.0%)	12 (3.0%)	0.024^*^
Signs and symptoms				
Fever days ≥3 days	847 (74.8%)	534 (73.7%)	313 (76.9%)	0.255
Cough	948 (83.7%)	593 (81.8%)	355 (87.2%)	0.022^*^
Sore throat	240 (21.2%)	146 (20.1%)	94 (23.1%)	0.275
Diarrhea	79 (7.0%)	41 (5.7%)	38 (9.3%)	0.027^*^
Dry throat	108 (9.5%)	63 (8.7%)	45 (11.1%)	0.232
Respiratory rate ≥ 30 times/min	47 (4.2%)	35 (4.8%)	12 (2.9%)	0.169
Oxygen saturation ≤ 93	369 (32.6%)	256 (35.3%)	113 (27.8%)	0.011^*^
Radiographically visible	1,130 (99.8%)	723 (99.7%)	407 (100.0%)	0.747
Oxygen absorption	947 (83.7%)	621 (85.7%)	326 (80.1%)	0.019^*^
High-flow oxygen absorption	781 (69.0%)	527 (72.7%)	254 (62.4%)	<0.001^*^
Respiratory failure and mechanical ventilation	234 (20.7%)	170 (23.4%)	64 (15.7%)	0.003^*^
Shock	24 (2.1%)	16 (2.2%)	8 (2.0%)	0.956
Other organ failure	39 (3.4%)	28 (3.9%)	11 (2.7%)	0.392
Diagnoses				
Severe	545 (48.1%)	360 (49.7%)	185 (45.5%)	0.195
Critical illness	287 (25.4%)	206 (28.4%)	81 (19.9%)	0.002^*^
Outcomes (%)				0.002^*^
Discharged	893 (78.9%)	549 (75.7%)	344 (84.5%)	0.001^*^
In hospital	35 (3.1%)	28 (3.9%)	7 (1.7%)	0.069
Death	204 (18.0%)	148 (20.4%)	56 (13.8%)	0.007^*^
In-hospital days [median (IQR)]	11.00 [8.00, 16.00]	12.00 [8.00, 17.00]	10.00 [7.00, 15.00]	0.003^*^

IQR, interquartile range; SD, standard deviation.

Value of *p*s comparing males and females are from the chi-square test or Mann–Whitney *U* test.

^*^Significant at *p* < 0.05.

**Table 2:** Sex-specific laboratory parameters of the cohort.

	All (*n* = 1,132)	Male (*n* = 725)	Female (*n* = 407)	value of *p*
White blood cell count [×10^9^/L, normal range 4–10, median (IQR)]	7.68 [5.54, 10.57]	7.73 [5.64, 10.66]	7.51 [5.51, 10.52]	0.474
Neutrophil count [×10^9^/L, normal range 1.8–6.3, median (IQR)]	5.00 [3.14, 7.48]	5.25 [3.39, 7.91]	4.73 [2.93, 7.00]	<0.001^*^
>6.3	410 (36.2)	281 (38.8)	129 (31.7)	0.021^*^
Neutrophil [%, median (IQR)]	71.27 [43.04, 87.78]	75.08 [45.87, 88.47]	66.15 [38.21, 84.88]	<0.001^*^
>75	311 (27.5)	225 (31.0)	86 (21.1)	<0.001^*^
Lymphocyte count [×10^9^/L, normal range 1.1–3.2, median (IQR)]	0.75 [0.49, 1.14]	0.70 [0.45, 1.06]	0.84 [0.56, 1.30]	<0.001^*^
<1.1	809 (71.5)	547 (75.4)	262 (64.4)	<0.001^*^
Lymphocyte [%, median (IQR)]	10.59 [5.66, 19.33]	9.74 [5.16, 17.86]	13.20 [6.62, 21.37]	<0.001^*^
<20	709 (62.6)	477 (65.8)	232 (57.0)	0.004^*^
Neutrophil/lymphocyte ratio [median (IQR)]	6.42 [3.16, 13.13]	7.10 [3.56, 14.34]	5.21 [2.83, 10.09]	<0.001^*^
Monocyte count [×10^9^/L, normal range 0.1–0.6, median (IQR)]	0.39 [0.26, 0.57]	0.41 [0.27, 0.57]	0.39 [0.26, 0.57]	0.382
Monocyte [%, median (IQR)]	4.72 [2.85, 7.36]	4.82 [2.90, 7.49]	4.57 [2.75, 7.08]	0.371
Hemoglobin [g/L, male normal range 130–175, female normal range 115–150, median (IQR)]	123.00 [108.00, 136.00]	127.00 [113.00, 139.00]	117.00 [103.00, 127.00]	<0.001^*^
Platelet count [×10^9^/L, normal range 125–350, median (IQR)]	185.00 [132.00, 245.00]	180.00 [125.25, 238.00]	193.00 [148.00, 260.00]	<0.001^*^
<125	241 (21.3)	174 (24.0)	67 (16.5)	0.004^*^
C-reactive protein [mg/L, normal range 0–10, median (IQR)]	23.53 [6.86, 82.60]	31.00 [8.60, 93.40]	15.80 [5.00, 60.80]	<0.001^*^
>10	672 (59.4)	464 (64.0)	208 (51.1)	<0.001^*^
Erythrocyte sedimentation rate [mm/h, male normal range 0–15, female normal range 0–20, median (IQR)]	48.00 [30.00, 75.00]	45.00 [30.00, 70.00]	50.00 [26.00, 82.00]	0.267
Procalcitonin [ng/ml, normal range 0–0.05, median (IQR)]	0.10 [0.05, 0.41]	0.11 [0.06, 0.42]	0.08 [0.04, 0.36]	0.023^*^
>0.05	439 (38.8)	311 (42.9)	128 (31.4)	<0.001
PH [normal range 7.35–7.45, median (IQR)]	7.42 [7.36, 7.45]	7.42 [7.37, 7.46]	7.41 [7.33, 7.45]	0.050^*^
Oxygen saturation [%, normal range 0–100, median (IQR)]	96.15 [92.00, 99.00]	96.00 [92.00, 98.05]	97.00 [92.15, 99.00]	<0.001^*^
<19.9	517 (45.7)	352 (48.6)	165 (40.5)	0.011^*^
Carbon dioxide partial pressure [mmHg, normal range 35–45, median (IQR)]	36.00 [31.77, 41.00]	36.00 [31.50, 41.00]	37.00 [32.00, 42.00]	0.503
Oxygen partial pressure [mmHg, normal range 80–100, median (IQR)]	89.20 [67.00, 136.00]	84.60 [64.00, 122.00]	103.75 [75.72, 161.25]	<0.001^*^
<80	307 (27.1)	230 (31.7)	77 (18.9)	<0.001^*^
D-dimer [ug/mL, normal range 0–0.5, median (IQR)]	1.17 [0.51, 3.66]	1.17 [0.50, 4.09]	1.13 [0.53, 3.23]	0.681
Prothrombin time [s, normal range 11–14, median (IQR)]	12.90 [12.15, 14.05]	13.10 [12.30, 14.20]	12.60 [11.80, 13.70]	<0.001^*^
>14	262 (23.1)	183 (25.2)	79 (19.4)	0.031^*^
<11	33 (2.9)	13 (1.8)	20 (4.9)	0.005^*^
Thrombin time [s, normal range 14–21, median (IQR)]	16.70 [15.80, 17.90]	16.70 [15.80, 17.80]	16.70 [15.90, 18.00]	0.986
Lactate dehydrogenase [U/L, normal range 135–225, median (IQR)]	257.65 [204.22, 344.38]	272.80 [209.00, 363.15]	236.20 [191.80, 313.30]	<0.001^*^
>225	557 (49.2)	388 (53.5)	169 (41.5)	<0.001^*^
Aspartate aminotransferase [U/L, normal range 0–36, median (IQR)]	30.50 [21.09, 45.38]	33.70 [23.88, 49.97]	25.62 [17.89, 38.47]	<0.001^*^
>36	396 (35.0)	288 (39.7)	108 (26.5)	<0.001^*^
Alanine aminotransferase [U/L, normal range 0–35, median (IQR)]	25.10 [16.60, 43.20]	28.20 [18.70, 48.30]	20.05 [13.88, 33.10]	<0.001^*^
>35	337 (29.8)	251 (34.6)	86 (21.1)	<0.001^*^
Total bilirubin [μmol/L, normal range 3–22, median (IQR)]	11.20 [8.60, 15.00]	12.19 [8.90, 16.00]	9.90 [7.60, 13.11]	<0.001^*^
>22	73 (6.4)	61 (8.4)	12 (2.9)	0.001^*^
Creatinine [μmol/L, normal range 46–92, median (IQR)]	73.20 [58.80, 99.20]	79.40 [64.73, 107.25]	61.90 [51.00, 81.80]	<0.001^*^
>92	296 (26.1)	227 (31.3)	69 (17.0)	<0.001^*^
Blood urea nitrogen [μmol/L, normal range 2.5–6.1, median (IQR)]	6.72 [4.86, 10.10]	7.29 [5.36, 10.85]	5.81 [4.40, 8.35]	<0.001^*^
>6.1	567 (50.1)	414 (57.1)	153 (37.6)	<0.001^*^
Creatine kinase [U/L, normal range 30–135, median (IQR)]	72.10 [42.60, 142.55]	86.20 [49.60, 184.38]	54.30 [34.50, 98.00]	<0.001^*^
>135	225 (19.9)	177 (24.4)	48 (11.8)	<0.001^*^
TNI [ng/ml, normal range 0–0.04, median (IQR)]	0.01 [0.01, 0.03]	0.01 [0.01, 0.04]	0.01 [0.00, 0.02]	<0.001^*^
>0.04	185 (16.3)	131 (18.1)	54 (13.3)	0.044^*^
Creatine kinase–MB isoform [ng/ml, normal range 0–4, median (IQR)]	1.90 [1.03, 4.18]	2.10 [1.10, 4.50]	1.80 [0.91, 3.70]	<0.001^*^
>4	259 (22.9)	179 (24.7)	80 (19.7)	0.063^*^
Myoglobin [ng/ml, normal range 0–70, median (IQR)]	52.80 [28.87, 120.62]	63.40 [34.20, 142.90]	39.60 [21.20, 74.90]	<0.001^*^
>70	353 (31.2)	266 (36.7)	87 (21.4)	<0.001^*^
B-type natriuretic peptide [pg/ml, normal range 0–100, median (IQR)]	105.20 [45.60, 253.10]	107.84 [46.08, 250.55]	103.00 [44.00, 281.40]	0.831
Natural killer cells [/μl, normal range 136–880/μl, median (IQR)]	109.00 [62.00, 182.00]	104.00 [60.00, 184.00]	114.00 [66.00, 175.00]	0.318
CD19 cells [/μl, normal range 92–498/μl, median (IQR)]	67.50 [36.00, 110.50]	64.00 [35.00, 103.00]	90.00 [40.00, 133.25]	<0.001^*^
CD3+ [%, normal range 52.11–82.55, median (IQR)]	59.73 [49.67, 70.05]	58.39 [48.61, 68.91]	62.20 [52.20, 70.85]	0.020^*^
CD3 + CD4 + CD8− [%, normal range 22.20–50.25, median (IQR)]	32.97 [25.52, 40.58]	32.09 [25.00, 39.69]	34.35 [27.26, 43.16]	0.017^*^
CD3 + CD16 + CD56+ [%, normal range 6.85–36.98, median (IQR)]	20.96 [12.41, 30.62]	22.26 [12.91, 32.84]	18.90 [11.33, 25.52]	<0.001^*^
CD3 − CD19+ [%, normal range 5.05–20.45, median (IQR)]	14.10 [8.49, 21.42]	13.36 [8.04, 20.46]	16.23 [9.65, 22.70]	0.016^*^
CD3 cells [/μl, normal range 834–2,217, median (IQR)]	320.00 [186.50, 518.50]	286.00 [173.00, 474.00]	394.50 [233.25, 626.50]	<0.001^*^
CD4 cells [/μl, normal range 395–1,264, median (IQR)]	174.00 [99.00, 306.50]	157.00 [89.00, 249.00]	209.00 [116.75, 403.75]	<0.001^*^
CD8 cells [/μl, normal range 269–1,059, median (IQR)]	100.00 [57.00, 181.50]	90.00 [52.00, 167.00]	122.00 [71.00, 208.00]	<0.001^*^
CD3 + CD4 − CD8+ [%, normal range14.19–43.41, median (IQR)]	18.72 [13.10, 26.50]	18.55 [12.66, 26.61]	19.00 [14.01, 25.75]	0.797
CD4/CD8 ratio [median (IQR)]	169.08 [104.16, 278.80]	171.92 [100.71, 278.43]	166.61 [114.60, 280.93]	0.322
Interferon *α* [pg/ml, normal range 0–8.5, median (IQR)]	2.44 [2.44, 3.82]	2.44 [2.44, 3.88]	2.44 [2.44, 3.32]	0.731
Interferon *γ* [pg/ml, normal range 0–23.1, median (IQR)]	2.44 [2.44, 3.58]	2.44 [2.44, 3.76]	2.44 [2.44, 3.19]	0.725
Interleukin 10 [pg/ml, normal range 0–12.9, median (IQR)]	2.44 [2.44, 3.23]	2.44 [2.44, 3.52]	2.44 [2.44, 2.55]	0.012 ^*^
Interleukin 12 [pg/ml, normal range 3.4, median (IQR)]	2.44 [2.44, 2.44]	2.44 [2.44, 2.44]	2.44 [2.44, 2.44]	0.779
Interleukin 17 [pg/ml, normal range 0–21.4, median (IQR)]	3.25 [2.44, 3.25]	3.25 [2.44, 3.25]	3.25 [2.44, 3.25]	0.742
Interleukin 1β [pg/ml, normal range 0–12.4, median (IQR)]	2.44 [2.44, 6.30]	2.44 [2.44, 6.30]	2.44 [2.44, 6.37]	0.630
Interleukin 2 [pg/ml, normal range 0–7.5, median (IQR)]	2.44 [2.44, 2.44]	2.44 [2.44, 2.44]	2.44 [2.44, 2.44]	0.887
Interleukin 4 [pg/ml, normal range 0–8.56, median (IQR)]	2.44 [2.44, 2.44]	2.44 [2.44, 2.44]	2.44 [2.44, 2.44]	0.691
Interleukin 5 [pg/ml, normal range 0–3.1, median (IQR)]	2.44 [2.44, 3.34]	2.44 [2.44, 3.29]	2.44 [2.44, 3.38]	0.832
Interleukin 6 [pg/ml, normal range 0–5.4, median (IQR)]	10.04 [2.99, 31.93]	12.77 [3.50, 37.57]	6.66 [2.44, 20.11]	<0.001^*^
>5.4	457 (40.4)	316 (43.6)	141 (34.6)	0.004^*^
Interleukin 8 [pg/ml, normal range 0–20.6, median (IQR)]	2.44 [2.44, 9.38]	2.44 [2.44, 11.04]	2.44 [2.44, 6.70]	0.599
Tumor necrosis factor *α* [pg/ml, normal range 0–16.5, median (IQR)]	2.44 [2.44, 2.44]	2.44 [2.44, 2.44]	2.44 [2.44, 2.44]	0.493
Aspartate aminotransferase/alanine aminotransferase ratio [median (IQR)]	1.24 [0.89, 1.77]	1.21 [0.87, 1.75]	1.28 [0.93, 1.79]	0.181
Blood urea nitrogen/creatinine ratio [median (IQR)]	0.09 [0.07, 0.11]	0.09 [0.07, 0.11]	0.09 [0.07, 0.11]	0.469

IQR, interquartile range; SD, standard deviation.

Value of *p*s comparing males and females are from the chi-square test or Mann–Whitney *U* test.

^*^Significant at *p* < 0.05.

### Outcomes

All patients were graded and defined according to the 9th Edition of the COVID-19 diagnosis and treatment plan issued by the National Health Commission, and critical patients included patients with respiratory failure requiring endotracheal intubation, patients with sudden shock, and patients with organ failure requiring ICU treatment.

### Predictive variables selection

All 1,132 patients hospitalized with Omicron were included in the variable selection and predictive models, and all of the collected variables were entered into the selection process. The outcome variables are critical illness, including shock (or death), ICU admission, and mechanical ventilation, and these three outcomes are severe outcomes of COVID-19. Critical illness has also been used to assess the severity of COVID-19 in previous studies (Gao et al., 2013; Metlay et al., 2019; Liang et al., 2020). A least absolute shrinkage and selection operator (LASSO) regression was used to select specific covariates in each sex to minimize the overfitting of variables and the potential collinearity of variables measured from the same patient. This regression penalizes the absolute magnitude of the coefficient of the regression model according to the value of lambda. The estimates of weaker factors shrank to zero for larger penalties, so that only the strongest predictive indicators would remain in the model, and most predictors were screened by the minimum value (λ min). The LASSO regression was conducted using the R package “glmnet” statistical software (R Foundation). In the process of data processing, we deleted the null values missing more than 50% in very few indicators and supplemented the null values missing less than 50% with the mean value of all other values. Subsequently, the final selected covariables were most frequently used in this screening process, and these significantly different variables screened separately were subjected to predictive models by multiple logistic regression for each sex. The optimal model is selected through multiple modeling after screening variables that are significantly different and consistent with clinical practice in the previous model. Finally, internal cross-validations of 200 bootstrap resamples were used to reduce overfit bias and for the accuracy estimates. To focus on the sex-specific effects of the selected potential difference features, a multiple logistic regression model was constructed in the whole sample, and the interaction between gender and difference features was analyzed. At the same time, a forest map was displayed based on the previous statistical results of the gender difference distribution.

### Statistical analysis

Continuous variables were represented by mean and standard deviation or median and quartile ranges (IQR) and analyzed using the Mann–Whitney *U* test. Categorical variables are expressed as counts and percentages for each category. Frequency comparisons of categorical variables were performed using the chi-square test and Fisher’s exact test, as appropriate. Candidate risk factors include age, sex, clinical symptoms, complications, laboratory results, and the number of people with abnormal results. A value of p less than 0.05 was considered statistically significant. In addition, the robustness of the resulting logistic regression model with varying numbers of selected covariates depends on quality measures, including specificity, sensitivity, and accuracy, as assessed by the area under the recipient–operator characteristic curve (AUC). All statistical analyses and data visualization were performed using R 4.2.1 (R Foundation, Vienna, Austria).

## Results

### Clinical characteristics of the cohort at the onset of admission

**Figure 1:**
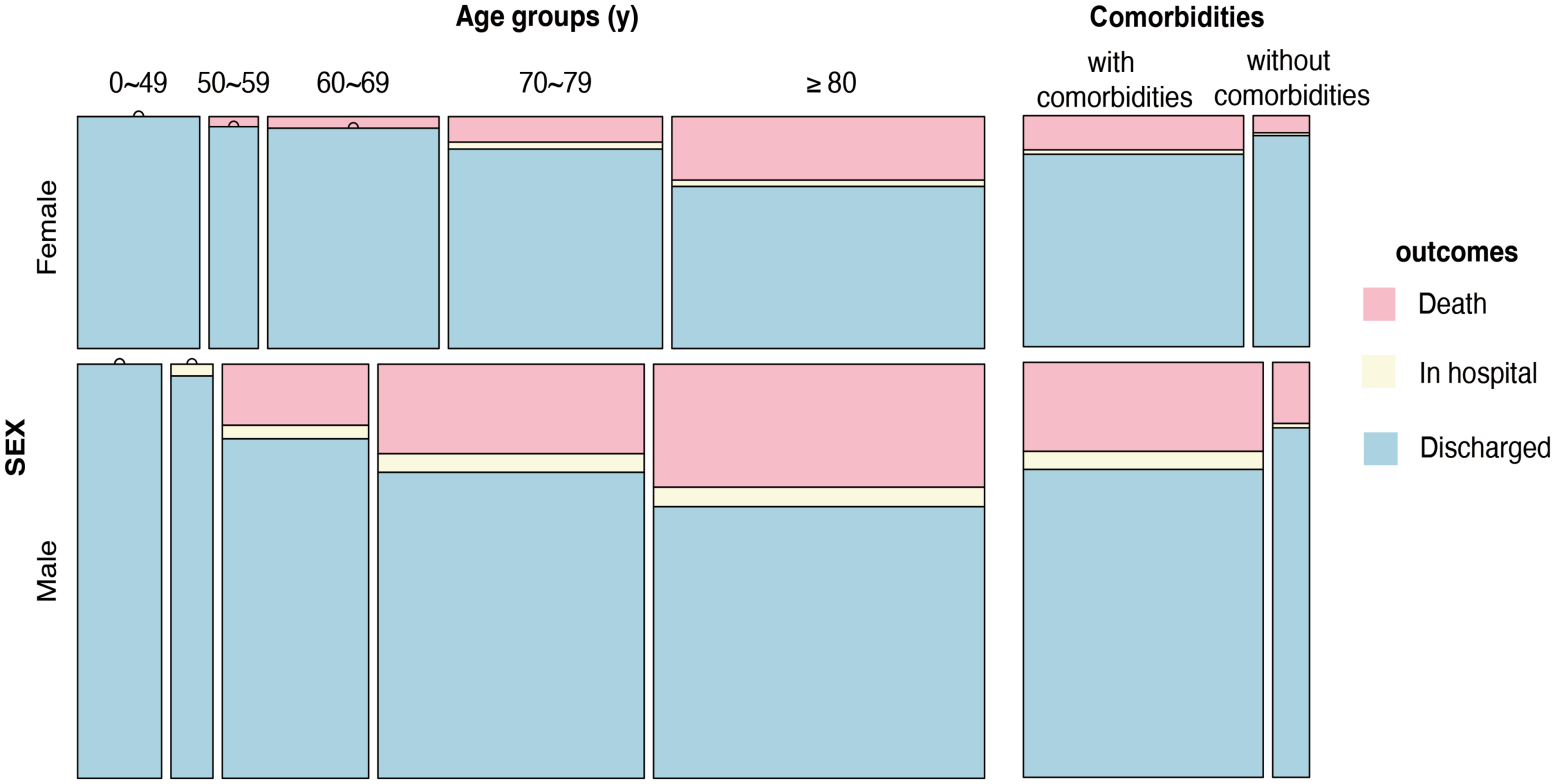
Sex-specific outcomes in all age groups, with or without underlying disease. Distribution of outcomes at different ages (0–49 years, 50–59 years, 60–69 years, 70–79 years, >80 years) and with or without comorbidities in males and females.

The general clinical characteristics of 1,132 nucleic acid- or antigen-confirmed COVID-19 cases in our study are shown in Table 1. Figure 1 illustrates sex-specific outcomes in all age groups, with or without underlying disease. Our retrospective study cohort comprised 725 males and 407 females. The median age in the male population was 71.7 years, the interquartile range (IQR) was 67.0–84.0, and the median age in the female population was 69.6 years (IQR: 63.25–84.75). There was no significant sex difference in age. Fever ≥3 days (74.8%), cough (83.7%), and sore throat (21.2%) were the most common symptoms, whereas diarrhea (7.0%) was rare. Notably, more females had cough (87.2% vs. 81.8%, *p* < 0.05) and diarrhea (9.3% vs. 5.7%, *p* < 0.05) symptoms than males. In addition, the days in the hospital were significantly different between males and females (12 vs. 10). Regarding comorbid conditions, males were more likely to have hypertension (56.1% vs. 48.4%, *p* < 0.05), cardiovascular disease (45.1% vs. 37.6%, *p* < 0.05), and basal lung disease (14.6% vs. 10.3%, *p* < 0.05), whereas females were more likely to have autoimmune diseases (3.0% vs. 1.0%, *p* < 0.05). Of the 1,132 patients with available data, 937 (84.1%) had at least 1 comorbidity. In addition, 287 patients (25.4%) were graded as critically ill, which showed sex-specific risk distributions of COVID-19 (*p* < 0.05), and the mortality of our Omicron variant-infected group was higher in males than in females (13.07% vs. 4.9%, *p* < 0.05). We also found that the underlying diseases were risk factors associated with the mortality of COVID-19 in our cohort for each sex, and both sexes displayed higher mortality rates with increasing age (shown in Figure 1).

### The sex-biased laboratory parameters of 1,132 hospitalized patients infected with the Omicron variant

All laboratory indices in Table 2 were measured at hospital admission. Of the 55 collected laboratory parameters, up to 32 showed significant differences between male and female patients, especially for functional indices of the liver, kidney, and inflammatory markers. Most of them were increased substantially in male patients, such as neutrophil count (NEU), neutrophil-to-lymphocyte ratio (NLR), platelet count (PLT), C-reactive protein (CRP), procalcitonin (PCT), prothrombin time (PT), aspartate aminotransferase (AST), alanine aminotransferase (ALT), lactate dehydrogenase (LDH), blood urea nitrogen (BUN), creatinine (CREA), total bilirubin (TIBL), creatine kinase–MB isoform (CK-MB), cardiac troponin I (cTnI), myoglobin (Myo), creatine kinase (CK), interleukin 6 (IL-6), and interleukin 10 (IL-10). However, oxygen partial pressure (PO_2_), oxygen saturation (SO_2_), lymphocyte count, and lymphocyte subsets such as CD3, CD4, and CD19 cells were substantially increased in female patients, as another study reported (*p* < 0.05, Table 2).

### Predictive sex-specific variable selection and the model construction of critical illness in each sex

**Figure 2:**
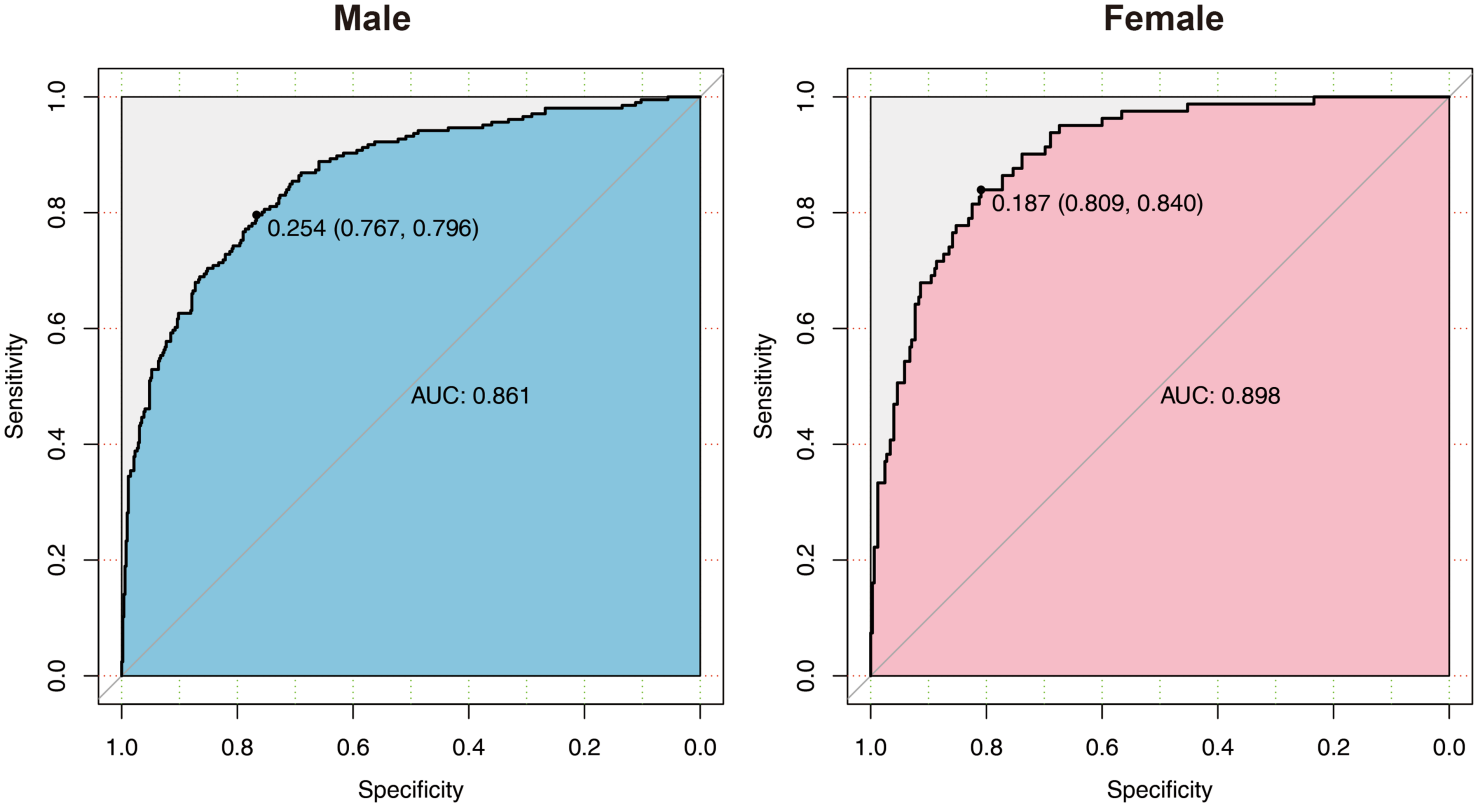
The ROC diagram of multiple logistic regression models for males and females. AUC, area under the curve.

All the variables in Tables 1, 2 were analyzed by LASSO regression in the male and female groups related to the critical illness outcome distinctively, and 46 significant predictors for males and 23 for females were screened, which were included in multiple logistic regression models. After the first multiple logistic regression modeling, we selected variables with significant differences in the models or significant differences at the edges for modeling again. Finally, we determined robust models for different sexes distinctively, and the AUCs of the models reached 0.861 and 0.898, respectively. For internal verification of the model (Tables 3 and 4), 200 rounds of 10-fold cross-validation found that the AUC of the model remained at 0.848 and 0.878 (Figure 2), indicating that the model was relatively robust.

**Table 3:** Male-specific multiple logistic regression model.

Male model						
	Estimate	Std. Error	*z* value	*p*	Wald	OR_with_CI
(Intercept)	−0.601	0.511	−1.174	0.240	1.379	0.548 (0.195 ~ 1.46)
RP ≥30 times/min	2.176	0.522	4.168	<0.001	17.371	8.813 (3.279 ~ 25.929)
BMI (body mass index)	−0.039	0.011	−3.601	<0.001	12.969	0.962 (0.941 ~ 0.982)
Neutrophil rate (%)	0.008	0.002	4.042	<0.001	16.339	1.008 (1.004 ~ 1.012)
Lymphocyte rate (%)	−0.031	0.011	−2.753	0.006	7.580	0.969 (0.946 ~ 0.989)
Hemoglobin (g/L, male normal range 130–175, female normal range 115–150)	−0.016	0.004	−4.073	<0.001	16.588	0.984 (0.976 ~ 0.992)
C-reactive protein (mg/L, normal range 0–10)	0.003	0.001	2.326	0.020	5.409	1.003 (1.001 ~ 1.006)
Carbon dioxide partial pressure (mmHg, normal range 35–45)	0.034	0.008	4.320	<0.001	18.663	1.035 (1.019 ~ 1.051)
Oxygen partial pressure (mmHg, normal range 80–100)	−0.008	0.003	−2.648	0.008	7.009	0.992 (0.985 ~ 0.998)
Lactate dehydrogenase (U/L, normal range 135–225)	0.003	0.001	4.580	<0.001	20.974	1.003 (1.002 ~ 1.005)
Myoglobin (ng/ml, normal range 0–70)	0.002	0.001	4.093	<0.001	16.755	1.002 (1.001 ~ 1.003)
Interleukin 6 (pg/ml, normal range 0–5.4)	0.001	0.001	1.017	0.309	1.034	1.001 (0.999 ~ 1.003)
Interleukin 10 (pg/ml, normal range 0–12.9)	0.060	0.033	1.801	0.072	3.244	1.062 (1.002 ~ 1.141)

**Table 4:** Female-specific multiple logistic regression model.

Female model						
	Estimate	Std. Error	*z*-value	value of *p*	Wald	OR_with_CI
(Intercept)	−3.946	0.682	−5.790	<0.001	33.522	0.019 (0.005 ~ 0.069)
Chronic liver disease	1.487	0.624	2.382	0.017	5.672	4.422 (1.286 ~ 15.333)
Fever days ≥3 days	0.776	0.417	1.863	0.063	3.470	2.173 (0.988 ~ 5.1)
Age ≥ 80 years	1.125	0.337	3.341	<0.001	11.160	3.08 (1.604 ~ 6.042)
BMI (body mass index)	−0.046	0.016	−2.818	0.005	7.942	0.955 (0.924 ~ 0.986)
White blood cell count (×10^9^/L, normal range 4–10)	0.067	0.026	2.523	0.012	6.364	1.069 (1.016 ~ 1.129)
Lymphocyte ratio (%)	−0.057	0.023	−2.439	0.015	5.951	0.945 (0.9 ~ 0.985)
Carbon dioxide partial pressure (mmHg, normal range 35–45)	0.023	0.008	2.982	0.003	8.890	1.023 (1.008 ~ 1.039)
Lactate dehydrogenase (U/L, normal range 135–225)	0.004	0.001	3.646	<0.001	13.293	1.004 (1.002 ~ 1.007)
B-type natriuretic peptide (pg/ml, normal range 0–100)	0.001	0.000	3.263	0.001	10.647	1.001 (1 ~ 1.002)
Interferon *α* (pg/ml, normal range 0–8.5)	0.046	0.025	1.842	0.066	3.392	1.047 (0.998 ~ 1.102)

**Figure 3:**
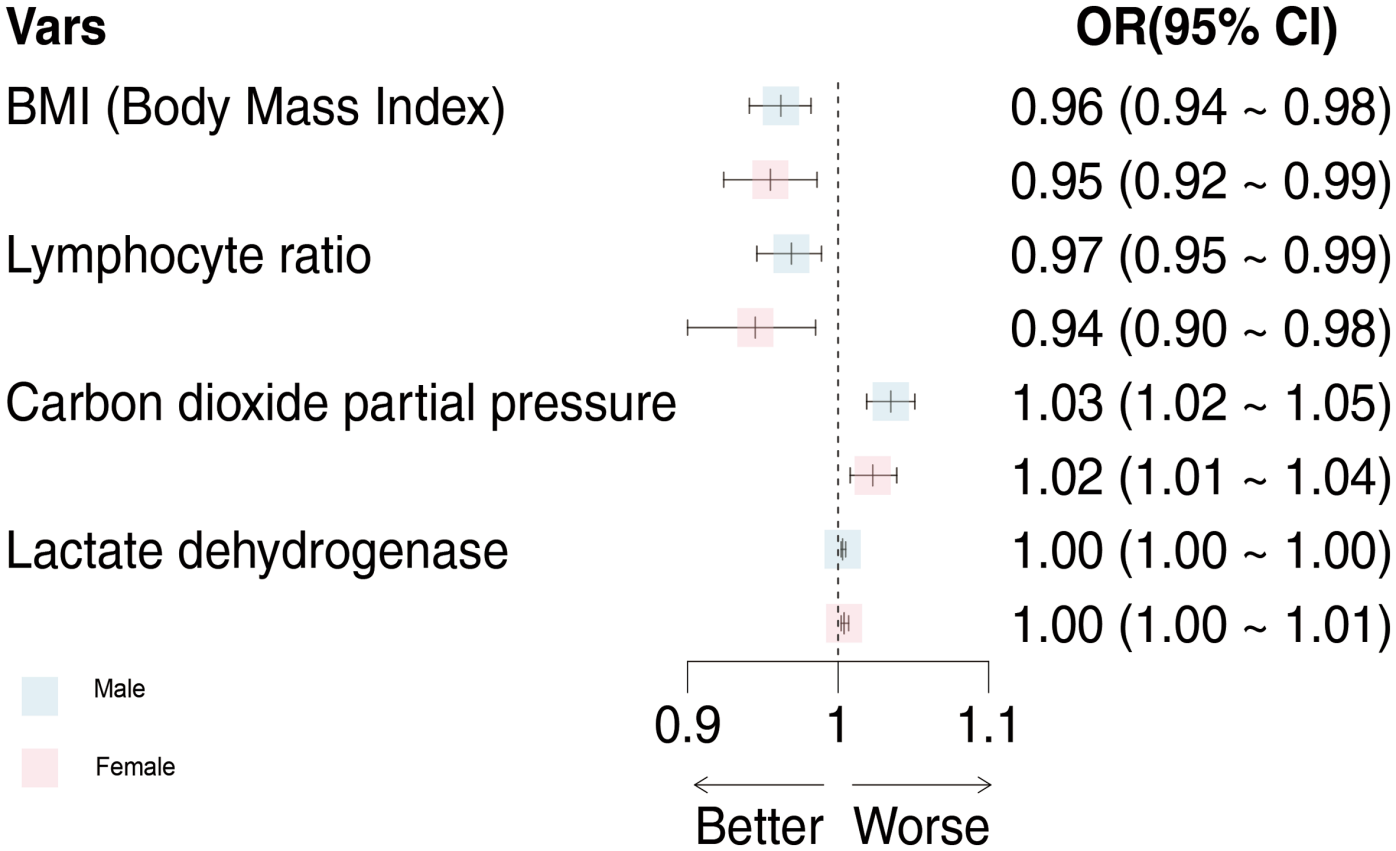
The effects of male and female models on critical illness were compared. OR, odds ratio; 95%CI, 95% confidence interval.

The common indicators in both models were BMI, LYM%, PCO_2_, and LDH, and we used a forest diagram to show the impact of four common variables on the outcome of critical illness (Figure 3). As seen from the figure, for any one of the common variables, the impact on critical illness is small. The predictors for critical illness in the male model also included RP ≥30 times/min (OR 8.813; 95% CI 3.279 ~ 25.929; *p* < 0.001), NEU (OR 1.008; 95% CI 1.004 ~ 1.012; *p* < 0.05), HBG (OR 0.984; 95% CI 0.976 ~ 0.992; *p* < 0.05), CRP (OR 1.003; 95% CI 1.001 ~ 1.006; *p* < 0.05), PO_2_ (OR 0.992; 95% CI 0.985 ~ 0.998; *p* < 0.001), MYO (OR 1.002; 95% CI 1.001 ~ 1.003; *p* < 0.001), IL-6 (OR 1.001; 95% CI 1.001 ~ 1.003; *p* = 0.309), and IL-10 (OR 1.062; 95% CI 1.002 ~ 1.141; *p* < 0.05). The model for females also included chronic liver disease (OR 4.422; 95% CI 1.286 ~ 15.333; *p* < 0.05), fever duration ≥3 days (OR 2.173; 95% CI 0.988 ~ 5.1; *p* = 0.06), age ≥ 80 years (OR 3.08; 95% CI 1.604 ~ 6.042; *p* < 0.001), WBC count (OR 1.069; 95% CI 1.016 ~ 1.129; *p* < 0.05), BNP level (OR 1.001; 95% CI 1.016 ~ 1.129; *p* < 0.05), and IFN-α level (OR 1.047; 95% CI 0.998 ~ 1.102; *p* < 0.05).

**Figure 4:**
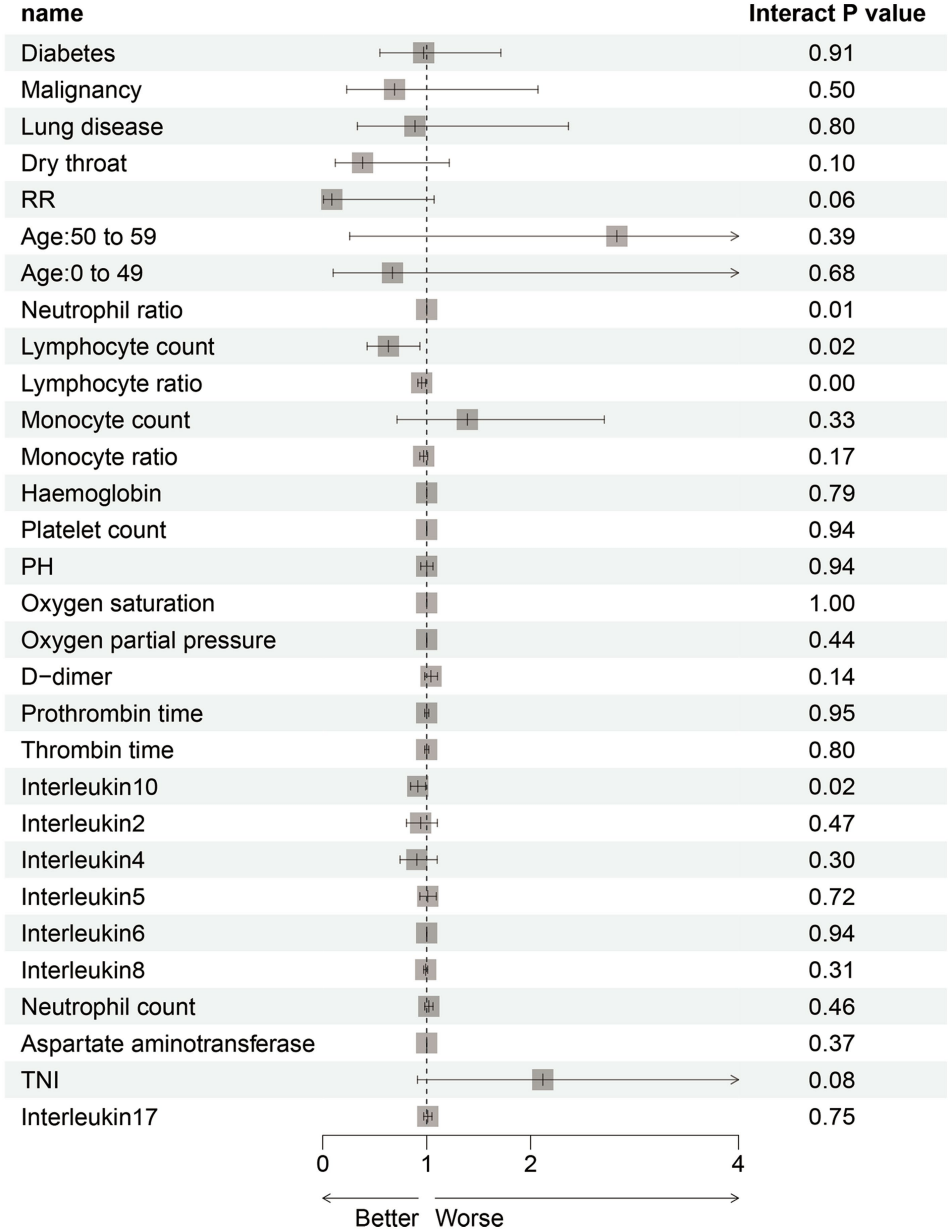
Interaction analysis of potential sex-specific characteristics and critical illness.

**Figure 5:**
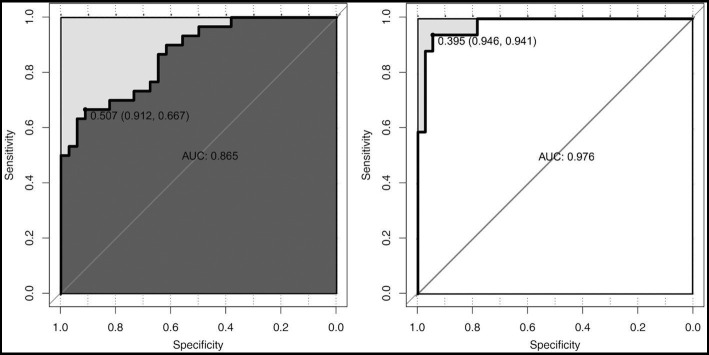
ROC diagram for male and female after matching.

To focus on the sex-specific effects of the selected potential difference features, another multiple logistic regression model was constructed for the whole cohort again, and the interaction between sex and specific indicators was analyzed. At the same time, the previous statistical results of the sex-specific distribution were combined. As a result, the interaction terms of four predictors (NEU, LYM, LYM%, and IL-10) were significant in the model, which indicated that there is a sex difference in the impact of critical illness outcome (Figure 4). In order to eliminate the doubt, we re-matched the data by age using propensity score matching to reduce the effect of confounding factors on our results. The re-matched 381 males and 381 females were re-analyzed using our sex-specific models. The results showed that the AUCs were 0.865 and 0.978 for males and females, respectively, which proved the validity of our sex-specific models and sex differences after 1:1 matching (Figure 5).

## Discussion

This study analyzed the sex differences in clinical characteristics and laboratory parameters in hospitalized Omicron patients and constructed predictive models for each sex group during the Omicron wave (5 November 2022 to 25 January 2023) in Shanghai, China. This was a rare, comprehensive analysis of sex differences in the COVID-19 Omicron variant. Our findings support the development and implementation of gender-specific and targeted prevention and treatment measures to minimize the adverse impact of the COVID-19 pandemic on human health. Since the first phase of the COVID-19 pandemic caused by the Delta and other prior variants, gender differences in incidence and outcome have been reported, with higher incidence among males identified from the beginning of the pandemic (Chen et al., 2020), although an earlier review has shown no gender difference in the absolute number of COVID-19 cases (Gebhard et al., 2020). Sex differences in COVID-19 have a common mechanistic basis, with both the innate immune system and the regulated renin–angiotensin system (RAS) involved. In addition, angiotensin-converting enzyme 2 (ACE2) is also involved in the pathogenesis of disease as a receptor for virus entry (Viveiros et al., 2021). One study found increased ACE2 in the lungs and hearts of aged males and a tendency for sex- rather than age-dependent patterns in the kidneys of males, which revealed organ-, sex-, and age-dependent differences in ACE2 regulation. These changes could lead to an increase in the severity and adverse outcomes reported in male COVID-19 patients. Their results also highlight that SARS-CoV-2 can target multiple organs other than the respiratory system, including the heart, kidneys, and intestines, mainly because ACE2 protein levels in these organs far exceed those in the lungs (Viveiros et al., 2022).

Our results are broadly similar to those previously reported among hospitalized cohorts. According to our study, there was no significant difference in age groups between males and females, but the incidence was higher in males (64.1%), and the risk of ICU admission or respiratory failure and mechanical intubation or death was almost two times higher among males than among females. Males over 60 showed worse outcomes than females, as reported in other studies (Gebhard et al., 2020; Jin et al., 2020; Stokes et al., 2020). Apart from death, males were found to have a higher length of stay (12 days vs. 10 days), which was also consistent with other reports (Vardavas et al., 2022).

Our results also showed that males had higher CRP, PCT, IL-6, and IL-10 levels and lower CD3+, CD4+, CD19+, and CD8+ cell levels than females. In addition, the difference between males and females is also reflected in the proportion of abnormal people in these indicators. Serum CRP and IL-6 levels are generally increased when bacteria, viruses, fungi, and other pathogens are infected, and high PCT levels usually indicate bacterial infection. These indicators can be used to predict mortality (Kiss et al., 2021). Notably, during SARS-CoV-2 infection, patients with COVID-19 experienced increased secretion or production of IL-6 and IL-8 and an overall decrease in CD4+ and CD8+, as well as T cells (Rabaan et al., 2021). Studies by Ruan et al. have shown that patients who died from COVID-19 had higher levels of IL-6 and ferritin than those who recovered (Ruan et al., 2020). Studies have shown that serum interleukin-10 levels are significantly higher in COVID-19 patients admitted to the intensive care units (ICUs) than in non-ICU patients. Importantly, elevated serum interleukin-10 levels in patients with COVID-19 infection may be both an anti-inflammatory mechanism and an immunosuppressive biomarker (Diao et al., 2020). Several valuable studies have found significant reductions in CD3+, CD4+, and CD8+ cells in non-survivors (Du et al., 2020; Liu et al., 2020), and due to their important role in viral clearance, these lymphocyte subsets reduce immune system overreaction.

Cytokine storms, which refer to the excessive production of various mediators caused by inflammation, have been identified as a major cause of critical illness and death from COVID-19 (Mehta et al., 2020). According to our research results, the value of these inflammatory indicators was significantly higher in males than in females and the proportion of abnormal indicators was also significantly higher in males, which directly leads to higher severity and mortality and rapid clinical progression to multiple organ failure and death in males, we hypothesized that early targeted inhibition of the release of associated inflammatory cytokines in elderly males infected with Omicron variants may be more effective in preventing worse outcomes. Inhibition of IL-6 receptors using tocilizumab in the treatment of cytokine storms in COVID-19 patients prevents severe complications of SARS-CoV-2 (Pelaia et al., 2021). Therefore, these inflammatory cytokines should be considered therapeutic targets to minimize the cytokine storm in males as soon as possible.

From our results, males also had a marked increase in NLR, PLT, PT, AST, ALT, LDH, BUN, CREA, TIBL, CK-MB, cTnI, Myo, and CK and a decrease in PO_2_, SO_2_, and LYM, which was consistent with the other variants analyzed before. As a systemic infection, inflammatory responses caused by COVID-19 infection could cause changes in peripheral blood cells and biochemical components (Tay et al., 2020), as well as all kinds of changes in different organs, such as the liver (Feng et al., 2020), kidney (Ronco et al., 2020), heart (Zheng et al., 2020), and gastrointestinal tract (Ng and Tilg, 2020). Our findings suggested that COVID-19 Omicron is more damaging to the liver, kidney, and heart in males. Therefore, timely targeted treatment in the case of abnormal indicators in male patients could effectively reduce the death of males caused by Omicron variant infection. The differences in PO_2_ and SO_2_ indicated that the damage to lung function caused by the Omicron variant was significantly different between the sexes, suggesting that early oxygen inhalation, high-flow oxygen inhalation, or even endotracheal intubation are also necessary for males.

More importantly, in view of the obvious gender difference in the Omicron variant, males and females were grouped, and variables related to critical illness (death, ICU admission, and mechanical ventilation) were screened out using lasso regression for modeling. After continuous optimization, we first constructed robust models for different sexes, and the AUCs of the different sex-specific predictive models reached 0.861 and 0.898, respectively. A forest diagram showed that the impact of any common variable on the outcome of a critical illness was limited. The predictors for critical illness in the male model also included other predictors, in which RP ≥30 times/min (OR 8.813; 95% CI 3.279 ~ 25.929; *p* < 0.001) had the most significant impact on critical illness. Chronic liver disease (OR 4.422; 95% CI 1.286 ~ 15.333; *p* < 0.05), fever days ≥3 days (OR 2.173; 95% CI 0.988 ~ 5.1; *p* = 0.06), and age ≥ 80 years (OR 3.08; 95% CI 1.604 ~ 6.042; *p* < 0.001) were more predictive of the critical illness outcome for the female model. Of course, the overall effect of the models for each sex might be more significant and predictive of critical illness. As part of the internal verification of the model, 200 times of 10-fold cross-validation found that the AUC of the model remained between 0.861 and 0.898. In addition, we re-matched all the data by age groups using propensity score matching to reduce the effect of confounding factors on our results, and it was proven that the sex-specific models before matching were still valid. Thus, it indicates that the models should be used by clinicians to estimate an individual hospitalized male’s or female’s risk of developing a critical illness. The establishment of gender-specific models and the screening and identification of gender-specific risk factors will help determine the clinical diagnosis and personalized treatment of sex differences in time to avoid delayed treatment and excessive waste of medical resources when the next wave of the epidemic comes.

## Limitations

As a prospective study, our laboratory results were tested according to the patients’ actual conditions, and incomplete data remains. In the process of data processing, we deleted the null values missing more than 50% in very few indicators and supplemented the null values missing less than 50% with the mean value of all other values. This may lead to some otherwise significant differences in the unscreened indicators.

Given the limitations, our study shows a higher risk for severe Omicron variant COVID-19 outcomes among males and created sex-specific predictive models distinctively. Sex is increasingly recognized as a modifier of disease, and its role in COVID-19 genomic variation appears to be no exception. Although the differences in immune response may be an explanation for sex-specific differences, further research is needed to identify more effective patient risk stratification and targeted treatment intervention strategies.

## Conclusion

Under the normal prevention and control of the novel coronavirus pneumonia epidemic, we found that the Omicron variant was similar to other variants, with significant gender and age differences in the clinical characteristics and laboratory indicators of critically ill patients. Based on this difference, we constructed critical illness prediction models of different genders so that clinicians could carry out critical illness assessments upon admission of COVID-19 patients while gaining precious time for the rescue of critically ill patients.

## Data availability statement

The original contributions presented in the study are included in the article/Supplementary material, further inquiries can be directed to the corresponding author/s.

## Ethics statement

The studies involving humans were approved by the Ethics Committee of Shanghai General Hospital. The studies were conducted in accordance with the local legislation and institutional requirements. Written informed consent for participation in this study was provided by the participants’ legal guardians/next of kin.

## Author contributions

JW: has access to all of the data in the study and takes responsibility for the integrity of the data and the accuracy of the data analysis, supervision, and obtained funding. JW and YZ: concept and design, administrative, technical, or material support. RY, XG, ZN, RZ, and SL: acquisition, analysis, or interpretation of data. RY and JW: drafting of the manuscript. RY, XX, and JW: statistical analysis. All authors: critical revision of the manuscript for important intellectual content.
