# Supplementary material for: Establishment of sex-specific predictive models for critical illness in Chinese people with the Omicron variant
Source: Front Microbiol. 2024 Jan 23;14:1224132. doi: 10.3389/fmicb.2023.1224132 (PMC10844546; doi:10.3389/fmicb.2023.1224132)
Supplement: Supplementary file 1 [file Table_1.docx]

**Table S1. Lineage distribution of the Omicron variants in 98 examined samples**

|  |  |
| --- | --- |
| **Lineage** | **Number** |
| BA.2 | 1 |
| BA.2.10.1  BA.2.75.5  BA.4.6  BA.5  BA.5.1  BA.5.1.5  BA.5.2  BA.5.2.1  BA.5.2.20  BA.5.2.28  BA.5.2.32  BA.5.2.34  BA.5.2.35  BF.4  BF.5  BF.7  BF.7.6  BF.7.1  BF.7.1  BM.1.1  BN.1.2  BN.1.3  BN.1.3.1  BN.3.1  BQ.1 | 7  1  1  1  1  1  16  2  1  1  1  1  1  1  2  28  8  5  1  3  5  2  1  4  1 |
| BR.2 | 1 |
